# Atypical Presentation of Majocchi's Granuloma in an Immunocompetent Host

**DOI:** 10.4269/ajtmh.16-0493

**Published:** 2017-01-11

**Authors:** Nisha V. Parmar, G. Johny Asir, Shivaprakash M. Rudramurthy

**Affiliations:** 1 Department of Dermatology, Venereology and Leprosy, Pondicherry Institute of Medical Sciences, Puducherry, India.; 2 Department of Clinical Microbiology, Pondicherry Institute of Medical Sciences, Puducherry, India.; 3 Department of Medical Microbiology, Centre of Advanced Research in Medical Mycology, World Health Organization Collaborating Centre, National Culture Collection of Pathogenic Fungi, Postgraduate Institute of Medical Education and Research, Chandigarh, India.

**Figure 1.**
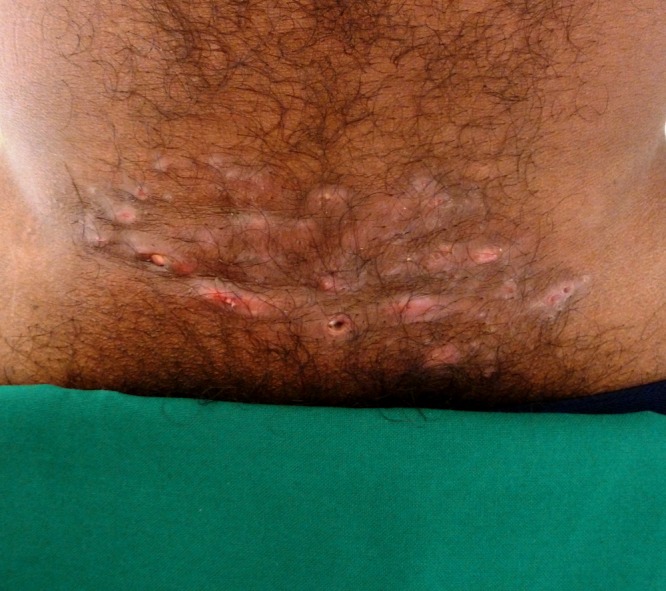
Clinical photograph showing multiple folliculocentric nodules with central pustulation on an area of 10 × 8 cm on the suprapubic area at initial presentation.

**Figure 2.**
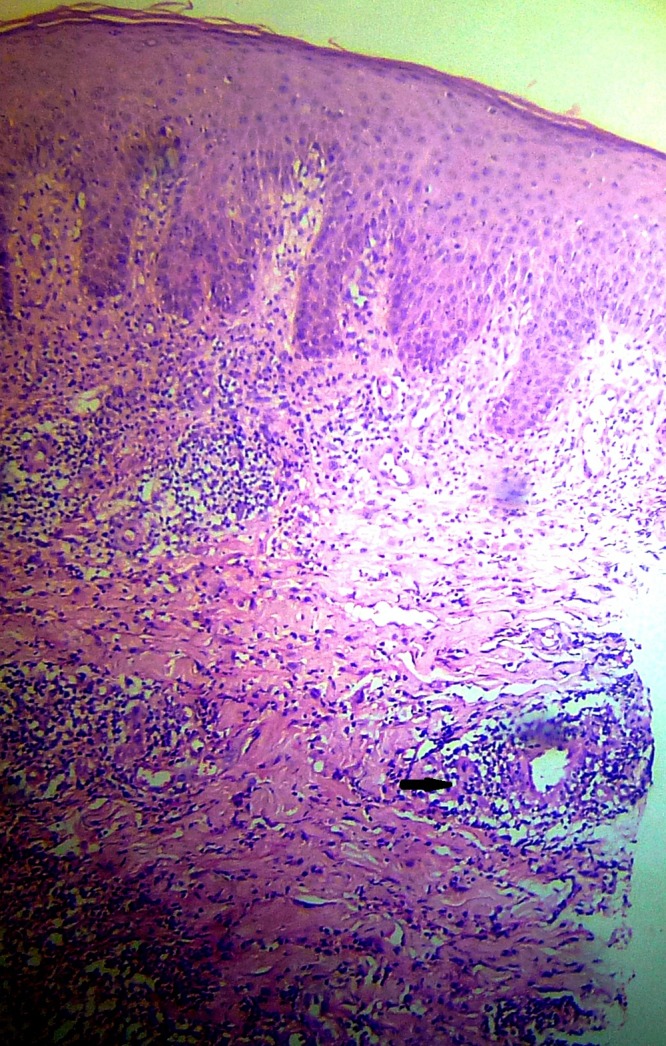
Skin biopsy of a nodule showing intense perifollicular lymphohistiocytic infiltrate (black arrow) in the dermis with destruction of hair follicles (hematoxylin and eosin ×100).

**Figure 3.**
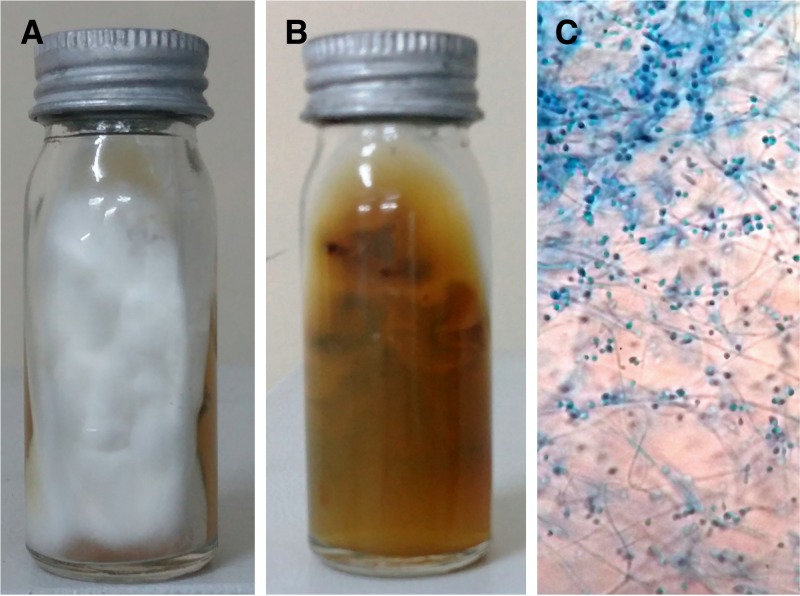
(**A**) Sabouraud's dextrose agar slant with white to cream colored velvety colonies with a flat topography (**B**) and a yellow to brown pigment on the reverse. (**C**) Lactophenol cotton blue mount (×400) showing thin hyaline, branching, septate hyphae with abundant spherical microconidia arranged in clusters; occasionally, slender macroconidia are seen.

**Figure 4.**
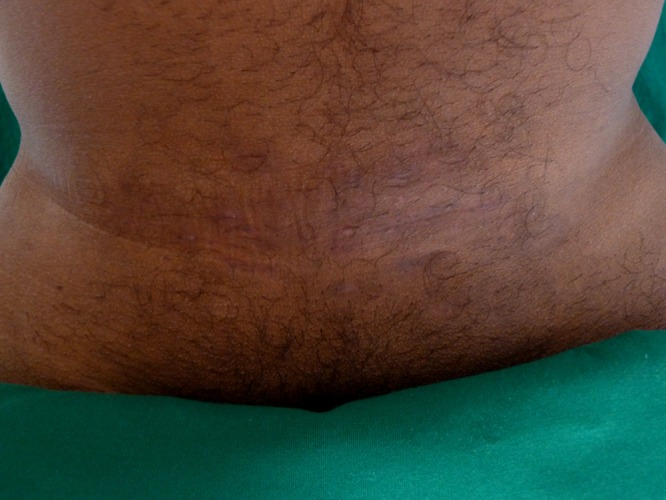
Clinical photograph showing complete clearance of lesions following 8 weeks of daily oral terbinafine.

A 42-year-old Indian man, farmer by occupation, presented to the dermatology outpatient department with complaints of itchy lesions on the lower abdomen of 2 weeks' duration. He was otherwise in good health. There was no history of trauma or application of topical corticosteroids over the affected area. He also denied shaving the area. General and systemic examinations were within normal limits. Cutaneous examination revealed multiple nontender erythematous folliculocentric nodules with central pustulation clustered on an area of 10 × 8 cm on the suprapubic area (Figure 1

). The clinical differential diagnoses considered were bacterial folliculitis, Majocchi's granuloma, actinomycosis, and tinea incognito. A Gram stain from the pustules showed numerous polymorphs but no bacteria. A 10% potassium hydroxide mount from a pustule revealed long branching septate hyphae. Other laboratory investigations including chest X-ray, fasting blood sugars, and liver and renal function tests were normal. Serologies for Human Immunodeficiency Virus, Hepatitis B and C viruses were nonreactive. Skin biopsy from one of the nodules revealed perifollicular lymphohistiocytic infiltrate with destruction of hair follicles (Figure 2

). Bacterial cultures were negative. Fungal cultures from the pus and skin biopsy specimen revealed dermatophyte growth, which was identified as *Trichophyton* sp. (Figure 3

) and sent to the National Culture Collection of Pathogenic Fungi, Postgraduate Institute of Medical Education and Research, Chandigarh, India. Based on the morphology and sequencing of the internal transcribed spacer (ITS) region of the rDNA, the isolate was confirmed as *Trichophyton interdigitale*. The ITS sequence had 100% similarity with the standard *T. interdigitale* strain, ATCC MYA-3108. The isolate is deposited at the center as NCCPF_800018. The patient was treated with oral terbinafine 250 mg once daily for 8 weeks. At 8 weeks' follow-up, there was complete resolution of the lesions (Figure 4

).

Majocchi's granuloma or fungal folliculitis is an uncommon presentation of dermatophytosis, described by Domenico Majocchi in 1883.^1^ Dermatophytes are keratinophilic fungi that infect the superficial layers of the epidermis. A breech in the epidermis paves way for the fungi to invade and reach the dermis where they elicit a florid inflammatory response due to their foreign nature. The commonest causative organism is *Trichophyton rubrum*.^2^ Two forms of Majocchi's granulomas are recognized. The follicular form occurs after trauma or chronic use of topical corticosteroids and is known to affect women who shave their legs. The subcutaneous nodular form is seen in immunocompromised hosts.^3^,^4^ This form can occur on any hair-bearing area of the body. Treatment of both the forms is with oral antifungal agents such as terbinafine or itraconazole for a prolonged duration, usually for 4–8 weeks.^5^

This case is being reported as it demonstrates the atypical location of Majocchi's granuloma in the suprapubic area in an immunocompetent host.
